# Dilatation of Urethra by the Urine

**Published:** 1878-03

**Authors:** H. B. Osborne

**Affiliations:** Sherman, N. Y.


SHERMAN, N. Y., Feb. 8th, 1878.

*Buffalo Medical and Surgical Journal:*

I notice in your last issue an article on "Dilation of the Urethra by the Urine itself." The article is one of great interest to me, as

I have been experimenting on several patients for the past four years and with wonderful results.

The *most important* clause of the treatment has been left out in the article mentioned, else I have made an improvement on the original plan, viz., that of attempting the ejaculation of urine while holding or compressing the urethral canal. The benefits accruing from this procedure are at once made plain by any person suffering from enlarged prostate, or with stricture, who adopts this plan of dilatation. In the winter of 1874 while spending a few weeks in New York, H. H., came to me saying that he had had stricture for ten years, and by advice of his physician had passed the sound thrice weekly for the past five years. He had discarded a No. 12 sound and had obtained a No. "9" which he had used for a year past and stated that he was sure the caliber of the urethra was growing less. On measarement I found that the urethra ought to have the caliber of "No. 18"—English seals. This measurement I based on the plan, or theory of Dr. Otis, and on trial I found two strictures, the first an inch from meatus caliber, of a No. 10 sound; second stricture two and a quarter inches from meatus which requires some force to pass the sound of No. 9 caliber. I requested him to wait until the bladder should become distended and then to grasp the penis between the thumb and finger and fill the urethra as full of urine as possible and then attempt ejaculation still holding and compressing the urethra firmly. Some six hours after, he came to my room looking pale and excited saying that he had attempted this procedure and that it had caused profuse hemorrhage and had become freightened because of it. On examination, I found that a No. 14 sound passed without any trouble. Thus reassured, I requested that he fill the urethra as before, every time that he was called upon to micturate, and to attempt ejaculation once daily. This he followed up for three months when I easily passed a No. 20 sound without pain. All trouble from the strictures he informed me disappeared some six weeks before. The gentleman wrote me some months since saying "that he remained well of his troubles yet he practiced the urine dilatation occasionally for fear of a return." I have treated three other cases with like favorable results, and feel enthusiastic over the matter.

Let any person, attempt this process who is not suffering from any urethral trouble, and he can at once realize something of the force which is brought to bear on the parts. It is sufficient to *rupture* the bands of hardened tissue as completely as any instrument yet devised for the purpose, and worthy of more serious consideration than has yet been given it.

H. B. OSBORNE, M. D.

